# A new endoscopic solution for rectal anastomotic atresia: Contrast agent-guided precision dilation

**DOI:** 10.1055/a-2239-3709

**Published:** 2024-02-22

**Authors:** Huawei Yang, Shaotong Wang, Rui Ji

**Affiliations:** 1 91623 Department of Gastroenterology, Qilu Hospital of Shandong University, Jinan, China

**Fig. 1:**
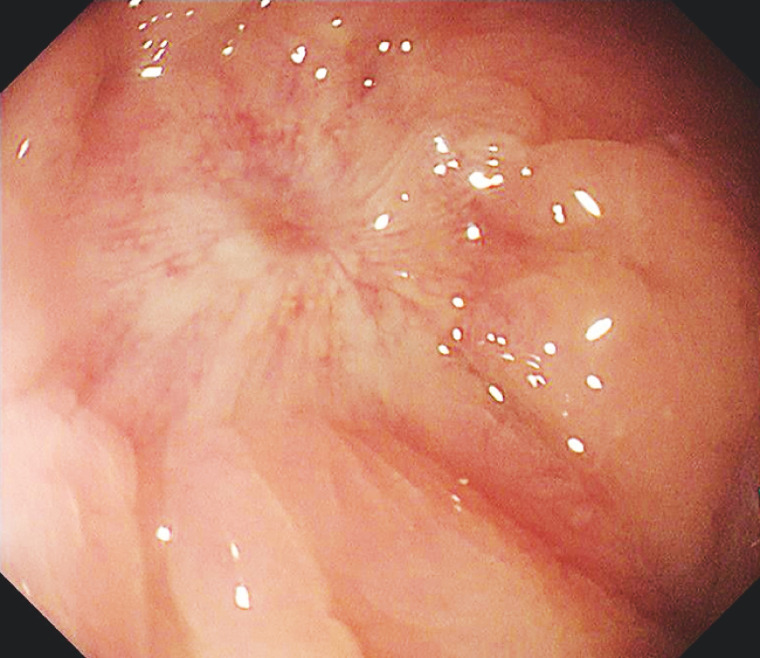
The rectal anastomosis is completely occluded.

**Fig. 2:**
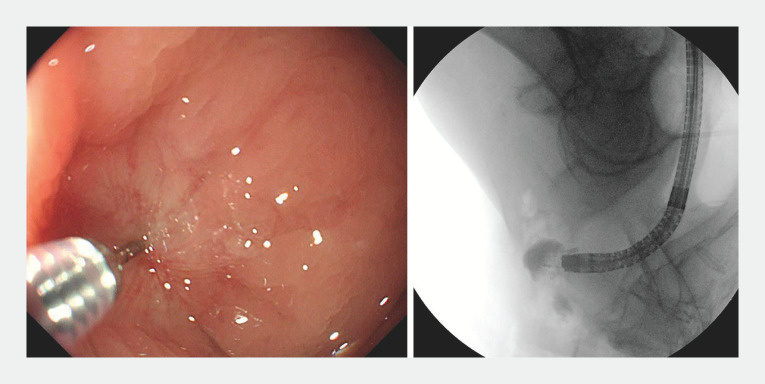
Injection needle puncture in the center of the atresia. X-ray fluoroscopy revealed the appropriate localization of the contrast medium within the distal intestinal lumen.

**Fig. 3:**
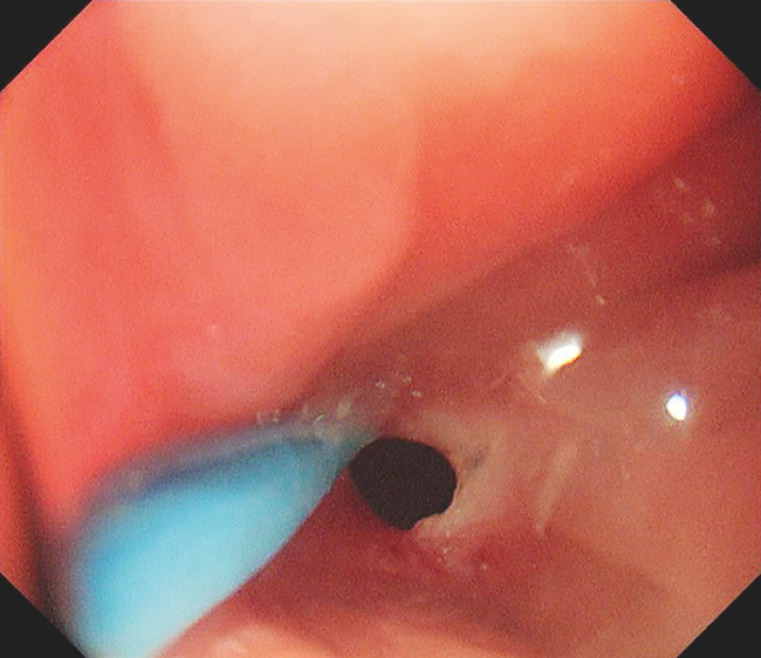
The anastomosis was opened by a small incision in the occlusion through the incision knife.

**Fig. 4:**
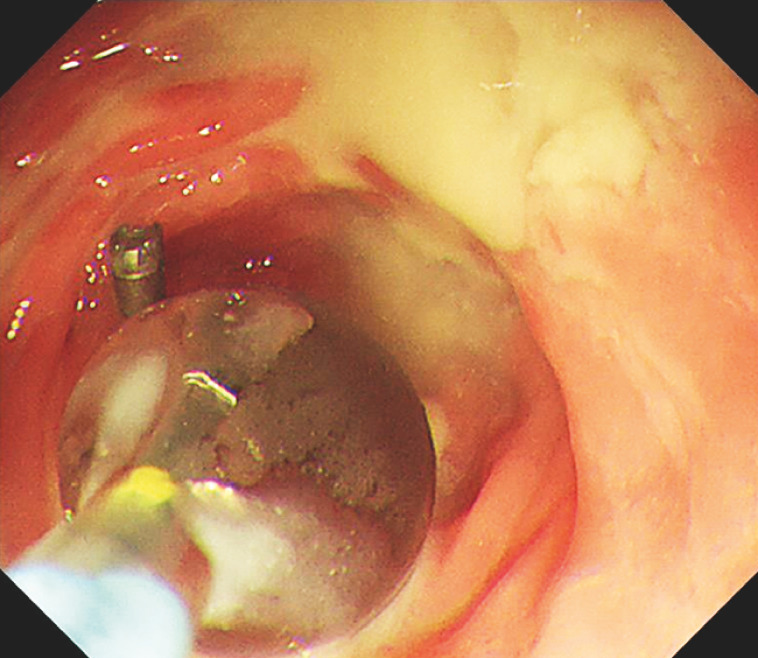
Follow-up colonoscopy in 2 weeks. A balloon 12 mm in diameter was inflated, effectively expanding the anastomosis.

**Fig. 5:**
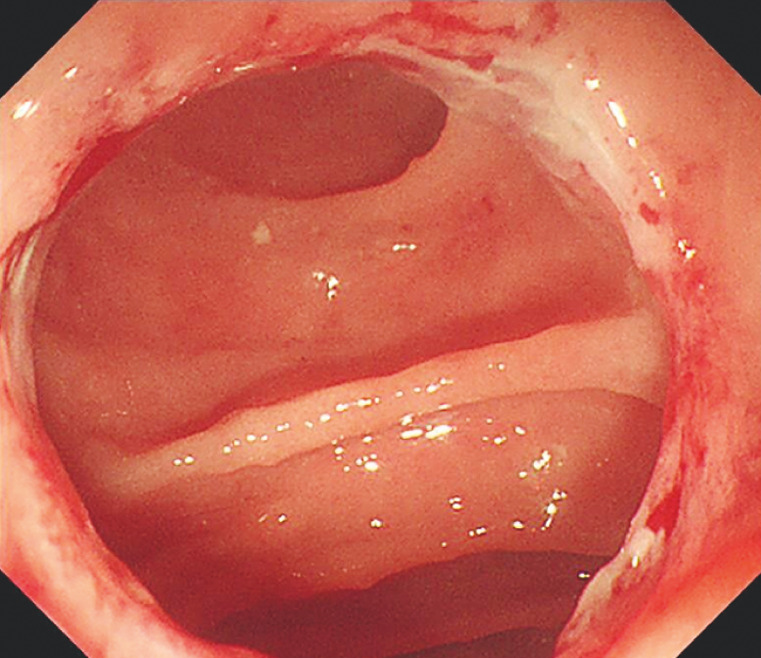
The endoscope passed easily through the anastomosis.

A 73-year-old man was admitted to our hospital due to rectal anastomosis atresia following prior treatments involving total colectomy, ileostomy establishment, and ileorectal end-to-side anastomosis for intestinal obstruction and ischemic necrosis approximately 4 months previously. Colonoscopy revealed occluded anastomosis 18 cm distal from the anus, characterized by the presence of white scarring and a prominent aggregation of mucosal folds at its central juncture ( Fig. 1 ). To precisely locate the proximal intestinal lumen, an injection needle (VedNeedle, 22G diameter; Vedkang Medical, Jiangsu, China) was used to penetrate the occlusion. After injecting the contrast medium, X-ray fluoroscopy revealed the appropriate localization of the contrast medium within the distal intestinal lumen ( Fig. 2 ). Guided by a guidewire (Jagwire, 0.035-diameter; Boston Scientific, Marlborough, Massachusetts, USA) through the incision knife, the anastomosis was then opened by a small incision in the occlusion, resulting in a controlled incision ( Fig. 3 ). An 8.0-mm biliary dilation balloon further opened the anastomosis successfully ( Fig. 4 ). After 2 weeks, a follow-up colonoscopy revealed anastomotic stenosis, which hindered the passage of an Olympus GIF Q260J scope (Olympus, Tokyo, Japan). To address this, a dilation balloon with a guidewire was introduced through the stenosis, and a 12-mm balloon was inflated, effectively expanding the anastomosis ( Fig. 5 ). The endoscope passed easily through the anastomosis ( Video 1 ). One month later, the patient made a positive recovery, leading to the successful ileostomy reversal procedure.

X-ray guided imaging after puncture needle is used to clarify the position of the distal intestinal lumen, after which incision and dilation are performed to treat a rectal anastomotic atresia.Video 1

Endoscopy_UCTN_Code_TTT_1AO_2AH

